# Perinatal intimate partner violence and postpartum contraception timing among currently married women in Southern Ethiopia: A multilevel Weibull regression modeling

**DOI:** 10.3389/fpubh.2022.913546

**Published:** 2022-10-19

**Authors:** Tafesse Lamaro Abota, Fikre Enqueselassie Gashe, Negussie Deyessa

**Affiliations:** ^1^College of Medicine and Health Sciences, Mizan-Tepi University, Mizan-Aman, Ethiopia; ^2^Department of Epidemiology and Biostatistics, School of Public Health, Addis Ababa University, Addis Ababa, Ethiopia

**Keywords:** perinatal, postpartum, contraception, multilevel, survival, Ethiopia

## Abstract

**Background:**

Adopting contraception on time is a critical intervention for postpartum women, but violence exposure around pregnancy may interfere with postpartum contraceptive use behaviors. Hence, this study aimed to investigate the time duration of the first modern contraceptive adoption and its individual-and community-level predictors among postpartum women in the Wolaita zone, South Ethiopia.

**Methods:**

A community-based prospective follow-up study was conducted among 1,292 postpartum women nested in 38 “*Kebles”* (clusters) using multistage-clustered sampling techniques. A multilevel Weibull regression model was employed to investigate predictors of time-to-method initiation after childbirth using STATA Version 14. Kaplan-Meier curve and Wilcoxon log-rank test were used to estimate time-to-modern contraceptive use across different variables. All variables with *p*-values <0.05 were considered for multivariate analysis. Adjusted time ratios (ATR) with 95 % CI were computed using Weibull accelerated failure time models.

**Results:**

Of the respondents, 62% (95% CI: 59.1–64.5) had started the first modern contraception within a year after childbirth. The restricted mean survival time-to-postpartum modern contraceptive use was 6.28 months. Being a rural dweller (aTR: 1.44; 95% CI: 1.06–1.99) and living in the middle household wealth quintiles (aTR: 1.10; 95% CI: 1.02–1.19) predicted longer time duration to adopt first modern contraception by 44 and 10%, respectively. The women from the community with a high early marriage (aTR: 1.14; 95% CI: 1.01–1.28) took longer time to initiate modern postpartum methods. Furthermore, women who had no history of perinatal abuse took less time than those who had a history of abuse to start postpartum contraception (aTR: 0.71; 95% CI: 0.66–0.78).

**Conclusion:**

Rural residence, poor household wealth status, history of perinatal abuse, and a high rate of early marriage in the community are predicted to lengthen the time duration to start modern postpartum contraception. Thus, community-level women's empowerment, particularly among rural women and integration of intimate partner violence screening into family planning counseling throughout the continuum of care will likely to improve postpartum contraception timing.

## Introduction

Intimate partner violence (IPV) is a global public health and human rights crisis that exacts a high burden of suffering on millions of women and families (1, 2). Violence against pregnant or postpartum women is a critical concern because of its pervasive impacts on several psychological and physical outcomes relevant to mother and child (3, 4). Perinatal IPV (P-IPV) refers to violence perpetrated by a partner either in the year before pregnancy, during pregnancy, or/and up to 1 year after childbirth (5, 6). Although perinatal women deserve safety and protection, violence during this critical period is associated with poor physical and psychosocial health, some of which may impact future childbearing and contraceptive use (7, 8). According to literatures, IPV has been linked to many reproductive health problems such as unintended pregnancies, lower contraceptive use, fetal loss, abortions, and a higher incidence of infertility (9–14). In terms of birth control, women in violent relationships have limited decision-making power. Studies have highlighted that women's ability to control their reproductive health choices significantly impacts greater control over pregnancy and pregnancy timing (15, 16).

Adopting contraception on time is a critical intervention for postpartum women who want to avoid unintended pregnancy and closed birth intervals (17, 18), leading to adverse maternal, perinatal and infant outcomes (19). The World Health Organization (WHO) recommends that women start modern methods immediately or within 42 days after childbirth, with the option of continuous contraception or effective switching for two subsequent years, depending on a woman's desire to space or limit future pregnancies (20, 21). Despite more than 90% of women want to avoid or delay pregnancies postpartum, two-thirds are not using contraception (22). Due to limited reproductive health control, women in abusive relationships are at a significantly higher risk of unintended pregnancy (8, 23, 24). Violence around the time of pregnancy may interfere with postpartum contraceptive use behaviors. The existing evidence reveals a variety of results (25). PIPV has been linked to lower or non-postpartum contraceptive use in some studies (13, 26–28), whilst other findings (29, 30) shows that PIPV exposure is associated with increased postpartum contraceptive adoption.

In recognition of its negative consequences, the family planning (FP) agenda 2030 aims to reduce psychosocial barriers that prevent women from using life-saving and life-changing modern contraceptives (31). Ethiopia has made significant progress toward meeting the FP2020 agenda, but the contraceptive prevalence rate remains low (32, 33). According to studies conducted in the country, about 46–66% of women initiated their first methods postpartum (34–36), whilst only 10–30% of them adopted within 2 months post-delivery (35, 36). Apart from this, the median survival time of the first modern contraceptive initiation was 7–11 months, far from the recommended time (35, 36). The timing of postpartum contraceptive adoption varies with an individual, partner, relationship, and community-level characteristics such as age, place of residence (37, 38), maternal education (35, 38), household wealth status (37), appropriate and timely maternal health care utilization (35, 36), breast feeding status (37), menstrual and sexual intercourse resumption and spousal communication (39). However, little is known about the effects of PIPV on time-to-modern contraceptive adoption among currently married women. Moreover, exploring how PIPV exposure affects postpartum women's contraceptive use patterns has important policy and program implications. Also, the finding will be critical in achieving national family planning program targets set in Health Sector Transformation Plan-II (Contraceptive prevalence rate from 41% in 2019 to 50% in 2025) (40). Therefore, this study investigates time duration-to-modern contraceptive adoption between postpartum women who had the experience of perinatal abuse and whom not, and to identify individual-and community-level predictors that influence postpartum women's method initiation in Wolaita zone, South Ethiopia.

## Materials and methods

### Study design, setting, and period

A community-based prospective follow-up study was conducted in the Wolaita zone located in Ethiopian's South Nations, Nationalities, and People's Regions (SNNPR). The zone is subdivided into sixteen rural districts (woredas) and six town administrations. It is one of the most densely populated zones in the region with an estimated population of 2.5 million people. The estimated number of women in the reproductive age group is 582,500. Of these women, the estimated postpartum population is 86,500. This zone has 310,454 households with an average household size of 4.84 persons (41). There are seven hospitals (five governmental and two private), 68 health centers, and 345 health posts within the zone (42). This study took place in randomly selected rural districts (Damot Woyde, Offa, Kindo Koysha, and Boloso Sore) and three town administrations (Soddo, Boditti, and Areka). The study was conducted between October 2019 and January 2021. The baseline data was collected between October 2019 and January 2020, and the final data was collected between October 2020 and January 2021.

### Source and study population with their eligibility criteria

All postpartum women living in the zone during the study period were considered the source population. The study population consisted of postpartum women in the zone's randomly selected districts and towns. The study sample's inclusion criteria were women of reproductive age, who lived with a current male partner for the last year, who were interviewed for baseline survey, and had a current healthy infant. The postpartum women who were divorced and widowed after the baseline survey were excluded from the study. The women who experienced miscarriages were also excluded from the final interview.

### Sample size determination

The sample size was computed using STATA Version 16.0. As no similar study was conducted in the country to determine the sample size; study from other developing country, Uganda (43) was used by taking 50% median survival time among groups, 90% power, 5% level of significance, and hazard ratio (1.83). By considering the design effect of 2 and 10% non-response rate, the final sample size was assumed to be 494. The following equations were used to calculate the required sample size. The sample size (n) = [*(*number of event/probability of event*)* ^*^ deff]/(1-non-response rate). Number of event $=\frac{{\left(Z_{\alpha /2+Z_{\beta }}\right)}^{2}}{pq{\left(\log AHR\right)}^{2}}$, where α = Threshold probability for rejecting the null hypothesis (Type I error rate), β = probability of failing to reject the null hypothesis under alternative hypothesis (Type II error rate), *p*= survival probability rate in group 1 (exposed), q = survival probability rate in group 2 (unexposed), and AHR = Adjusted hazards ratio. The probability of event = Pr (event) = 1 – [P S1 (*t*) + q S2 (*t*)]. However, this study was part of a large longitudinal study that designed to investigate the interplay between PIPV and postpartum contraception. The study had four specific objectives, and the alternative sample size for each was calculated. Thus, maximum sample size (1320) was taken for all objectives considering the following assumptions: 95% confidence interval, 4% margin of error, 80% power, proportion of postpartum modern contraceptive use (49%) (38), design effect of 2, and 10% non-response rate. The following single population proportion formula was used to calculate the sample size. *N* = [*{(Z*α*/2)*^2*^p (1–p)}/d^∧^2^*^deff]/ (1-non-response rate), where *z* = percentile of the standard normal distribution, *p* = proportion of postpartum modern contraceptive adoption from the other study, d = the desired precision of the estimate, deff = design effect for the multi-stage nature of the sampling procedure. However, to increase the study's power, 1,342 postpartum women who met inclusion criteria were approached at the baseline interview. After excluding non-response cases, 1,292 women had been followed for a year after the baseline survey. As a result, all 1,292 women were included in this study.

### Data collection procedure

An interviewer-administered questionnaire was prepared from different literature including WHO and DHS standard tools (44, 45). The pilot study was conducted to test questionnaire's validity and reliability and some modifications were made including frequency and timing of violence occurrence in the perinatal period (before, during and after pregnancy). As this was part of a large longitudinal study, the data were collected in two phases. At the baseline, socio-demographic, economic, psychosocial, and reproductive characteristics were collected. Then, participants had been on follow-up for a year and data on the reproductive events (e.g., breastfeeding, resumption of menses and sexual activity), PIPV exposure status, and contraceptive use dynamics (adoption, switching, continuation, and discontinuation) were collected at the final interview. Thirty-eight data collectors (married, female, diploma holders) with eight supervisors (BSc in Public Health) were trained and deployed after receiving 2 days of intensive training. For administrative purposes, the training was given separately in each district. The main focus of the training was on the purpose of the study, the contents of the instruments, and how to check the nuances of coding, errors, and consistency of each questionnaire.

### Sampling procedure

A multistage-clustered sampling technique was used to identify study participants. The zone is divided into sixteen rural districts and six town administrations. As the rule of thumb (>25%), four rural districts and three town administrations were randomly selected. These districts and towns were further clustered by “Kebles,” Ethiopia's lowest administrative unit, and stratified into rural and urban Kebles. In this study, a cluster is a community of people likely to share common values. Then, four rural and two urban Kebles were randomly selected from each rural district. Fourteen Kebles were chosen from town administrations (eight urban and six rural) using a simple random sampling method. With this, thirty-eight (twenty-two rural and sixteen urban) Kebles were drawn from randomly selected districts and towns. The sample size for each Keble was allocated using probability proportional to the size and the expected number of postpartum women per Keble. List of households with eligible women were prepared from a family folder of health extension workers (HEWs) in the respective Kebles. Enumerators compiled the lists with the help of HEWs. When there was more than one eligible woman in a household, only one woman was chosen randomly. Finally, 1,342 eligible women who met the eligibility criteria were included in the baseline survey. However, 1,292 women had been on 1 year follow-up for this study.

### Study variables and measurement

The outcome variable was time length-to-modern contraceptive use postpartum. This was recorded in months using a contraceptive calendar (46, 47). The event's occurrence was coded as “1” when women report modern contraception adoption and “0” otherwise. PIPV (psychological, physical, and sexual violence) was the main exposure variable measured using section seven of the WHO standardized questionnaire (44). Overall, the experience of PIPV was classified as a binary variable (yes/no). The women-level predictors included twelve variables that consisted of women's age at childbirth and marriage, education, employment status, number of living children, breastfeeding status, resumption of menses, attitudes toward wife-beating norms, exposure to perinatal violence (before, during pregnancy or either), and women's wealth status. The five husband-level predictors included education and employment status, alcohol and substance abuse history, and wife controlling behavior. The relationship-level predictors incorporated were women's decision-making autonomy, asset ownership, couple's communication about daily life, and income difference. Women's norms and attitudes toward IPV and a man's control over his wife's behaviors and activities were measured using sections six and seven of the WHO multi-country study on women's health and domestic violence questionnaire (44, 45). Participants' decision-making autonomy in household issues was also measured (45) by asking whether women participated in personal health care, daily household purchases, major household purchases, visits family or relatives, husband's and her income.

Community-level variables included were women's residency; classified as urban or rural based on the Ethiopian Central Statistical Authority descriptions of respondent's location (48). Aggregating individual-level characteristics constructed other community-level factors. The aggregates for clusters were computed using means (for normally distributed) or median (not normally distributed) values for each respondent in each category of a given variable. Finally, high-level variables were re-categorized into lower and higher categories.

### Data management and analysis

The data were coded, cleaned, and edited using SPSS for Windows version 25.0. Descriptive and summary statistics were computed in number and percentages. Multilevel survival models based on different parametric distributions were fitted because the hierarchical nature of data collected from 1,292 postpartum women nested in 38 clusters (Kebles). The study participants within each cluster ranged from twenty to forty-three. The multilevel survival model is the best model for the right-censored data and yields unbiased estimates of the risk of the occurrence of the target event (49). Consequently, the model handles the cluster-specific random effects on the survival outcomes (50). The effect of covariates on baseline hazards function is measured through two often-used models: the accelerated failure-time (AFT) model and the PH model. The covariate effect is multiplicative on the time scale in the AFT model, while it is multiplicative on the hazard scale in the PH models (50, 51). We preferred AFT to the PH model; hence it accounts for the effect of the covariates directly on the survival times rather than on the hazards rate as in the PH model, and it yields more accurate inference, proper fitting of the model and easy interpretation of the results (52, 53). Then, time ratios rather than hazard ratios were used to report time length-to-postpartum contraceptive use. The intraclass correlation coefficient (ICC) for the intercept only model was calculated to determine whether or not the multilevel survival analysis was required. ICC measures the total variation of postpartum contraceptive use timing between clusters without any covariates (52, 54). The model comparison was made using the log-likelihood ratio test, deviance (-2LL), and Akaike's Information Criterion (AIC) value. The model with the lowest deviance and AIC was selected as the best fitted model and used to describe the data. All variables with a *p*-value of <0.05 in bivariate analysis were considered candidates for multivariable analysis. In the multivariable multilevel analysis, the adjusted time ratios along with the 95% CI were used to show level of significance and strength of association.

### Ethical consideration

The study was reviewed and approved by the Institutional Review Board of the College of Health Sciences, Addis Ababa University, with a protocol number of 006/19/SPH. The interviews were conducted with full respect for WHO ethical and safety recommendation guidelines (55). All the study participants were briefed about the aim and procedures of the research and their right to abstain or withdraw from the study at any time. The informed verbal consent was obtained from each participant separately. The confidentiality of the collected data was maintained by locking it in the file cabinet. All study information was kept secured and confidential with the first author. After the interview, participants were allowed to visit a psychiatric nurse if they experienced any psychological discomfort.

## Results

### Individual- and community-level characteristics of study participants

A total of 1,252 of the study participants had completed the interview. About 3.1% of them were lost to be reached and censored for survival analysis (Table 1). The majority of the respondents were aged 25–34 years (57.1%), had no formal education (36%), were married to men with no education (30%), unemployed (85%) and had husbands who work in paid jobs (35%). Approximately 64% of the respondents reported resumption of menstruation, and 95% of them resumed sexual activity in the year postpartum. About 57% of the participants had justified IPV favoring norms, and 38% of the participants reported being exposed to violence in the year before pregnancy with 28% of them experienced it during pregnancy. Approximately 40% of women experienced perinatal partner violence either a year before or during pregnancy. Regarding community-level characteristics, the majority of respondents were living in the community with rural residence (56.3%), low early marriage (52.8%), high female literacy (55.7%), high IPV favoring norms (53.2%), and high women's decision-making autonomy (54.3%).

**Table 1:** Individual (women, partner and relationship) and community-level characteristics of study participants (*n* = 1292).

**Survival status**					
**Variables**	**Category**	**Failures(contraceptive users)** ***n*** = **776**		**Censored (nonusers)** ***n*** = **516**	
		***n***	***%***	***n***	***%***
**Woman level factors**					
Maternal age– (years)	≤24	149	19.2	146	28.3
	25–34	468	60.3	270	52.3
	35–49	159	20.5	100	19.4
Maternal age at marriage	<18 years	230	29.6	169	32.8
	≥18 years	546	70.4	347	67.2
Maternal education	Illiterate	257	33.2	205	39.8
	Primary	244	31.4	157	30.4
	Secondary +	275	35.4	154	29.8
Maternal employment status	Not employed	636	82.0	463	89.7
	Employed	140	18.0	53	10.3
Number of living children	1–2	316	40.7	217	42.1
	3–4	276	35.6	189	36.6
	≥5	184	23.7	110	21.3
Breastfeeding status	No	460	59.3	321	64.6
	Yes	316	40.7	176	35.4
Resumption of menses	No	168	21.6	302	58.5
	Yes	608	78.4	214	41.5
Justify wife-beating norms	No	359	46.3	186	36.0
	Yes	417	53.7	330	64.0
Abuse before the index pregnancy	No	536	69.1	273	52.9
	Yes	240	30.9	243	47.1
Abuse during index pregnancy	No	601	77.4	324	62.8
	Yes	175	22.6	192	37.2
Abuse during or after pregnancy	No	525	67.7	256	49.6
	Yes	251	32.2	260	50.4
Wealth Status	Poor	132	25.6	167	21.5
	Middle	264	51.2	409	52.7
	Rich	120	23.3	200	25.8
**Partner level factors**					
Employment status	Non-employed	489	63.0	347	67.2
	Employed	287	37.0	167	32.8
Educational Status	Illiterate	221	28.5	167	32.4
	Primary	224	28.9	145	28.1
	Secondary +	331	42.7	204	39.5
Alcohol misuse	No	535	68.9	359	69.6
	Yes	241	31.1	157	30.4
Substance abuse	No	668	86.1	448	86.8
	Yes	108	13.9	68	13.2
partner controlling behavior	No	409	52.7	202	39.1
	Yes	367	47.3	314	60.9
**Relationship level factors**					
Decision-making autonomy	No	416	53.6	308	59.7
	Yes	360	46.4	208	40.3
Duration of marriage	1–5 Years	227	29.3	176	34.1
	6–10 Years	307	39.6	193	37.4
	≥11 Years	242	31.1	147	28.5
Couple communicate daily life	No	225	29.1	205	39.7
	Yes	550	70.9	311	60.3
Asset Ownership (*n* = 764)	No	289	63.9	217	69.6
	Yes	163	36.1	95	30.4
Couple income difference	No income	475	61.2	335	64.9
	Earns less	199	25.6	131	25.4
	Earns more	102	13.1	50	9.70
**Community-level characteristics**					
Place of residence	Urban	253	45.5	212	41.1
	Rural	423	54.5	304	58.9
Early marriage	Low	433	55.8	249	48.3
	High	243	44.2	267	51.7
Community- level women literacy	Low	350	45.1	222	43.0
	High	426	54.9	294	57.0
Community norms favoring IPV	Low	382	49.2	223	43.2
	High	394	50.8	293	56.8
Decision-making autonomy	Low	347	44.7	243	47.1
	High	429	55.3	273	52.9
Place of delivery	Facility	598	77.1	253	49.0
	Home	178	22.9	263	51.0
Wealth Status	Poor	254	32.7	162	31.4
	Middle	258	33.2	181	35.1
	Rich	264	34.1	173	33.5

### Postpartum women's contraceptive use dynamics in the Wolaita zone, Southern Ethiopia

Of the study participants, 62%(95% CI: 59.1%, 64.5%) had started the first postpartum modern contraception in the year postpartum (Table 2). Injectables (44.1%), pills (16.9%), and Implant (15.3%) were the most commonly used modern methods. More than one-third (33.1%) of women discontinued their first modern contraception after childbirth, and 57.6% did not use any methods after discontinuation. At the time of the survey, nearly half of the postpartum women were using contraceptives. Injectable was the most preferred method (47.2%), followed by Implants (25.7%) and IUCD (14.4%), with half (51.3%) reporting side effects from the current method, the majority of respondents (27.0%) stated that they intended to discontinue current methods.

**Table 2:** Postpartum women's contraceptive use dynamics among currently married women in Wolaita zone, South Ethiopia.

**Variable**	**Measurements**	***n***	***%***
Have you used the first modern methods after childbirth	No	476	38.0
	Yes	776	62.0
Type of modern methods used after childbirth (*n* = 776)	Tubal litigation	1	0.1
	IUCD	91	11.7
	Implanol	119	15.3
	Injectables	342	44.1
	Pills	131	16.9
	Condom	34	4.4
	Emergency contraception	40	5.2
	Others	18	2.3
Are you currently using first methods	No	257	33.1
	Yes	519	66.9
Have you used any method after discontinuing (*n* = 257)	No	148	57.6
	Yes	109	42.4
Did you discuss with your husband before discontinued	No	112	43.6
	Yes	145	56.4
Was your husband forced you to discontinue	No	137	53.3
	Yes	120	46.7
Who initiated the methods discontinuation	Women	148	57.6
	Husband	89	34.6
	Third body	20	7.8
Are you currently using any methods	No	634	50.6
	Yes	618	49.4
Type of current methods you have been using	Tubal litigation	2	0.3
	IUCD	89	14.4
	Implanol	159	25.7
	Injectables	292	47.2
	Pills	62	10.0
	Condom	3	0.5
	EC	5	0.8
	Others	6	0.1
Have you been experiencing side effects for the current methods	No	301	48.7
	Yes	317	51.3
What measures are you currently taking for the side effects (*n* = 230)	Making home remedies	33	14.3
	Trying to consult HCPs	43	18.7
	Get advice from friends	9	3.7
	Get advice from husband	32	13.9
	Want to change the method	51	22.2
	Want to stop the method	62	27.0
Were you told by health professional about the side effects	No	147	46.4
	Yes	170	53.6
Who initiated the current methods	It is me	316	51.1
	It is my husband	76	12.3
	Jointly	226	36.6
Reasons for not using the methods currently (*n* = 634)	Breastfeeding	56	8.8
	Postpartum abstinence	30	4.7
	Not resumed menses	96	15.1
	Advised from HP	17	2.7
	Husband not wanting	185	29.2
	Feared side effects	172	27.1
	To be become pregnant	63	9.9
	Others	15	2.4

### Survival analysis result for time interval-to-modern contraception adoption among postpartum women

**Figure 1:**
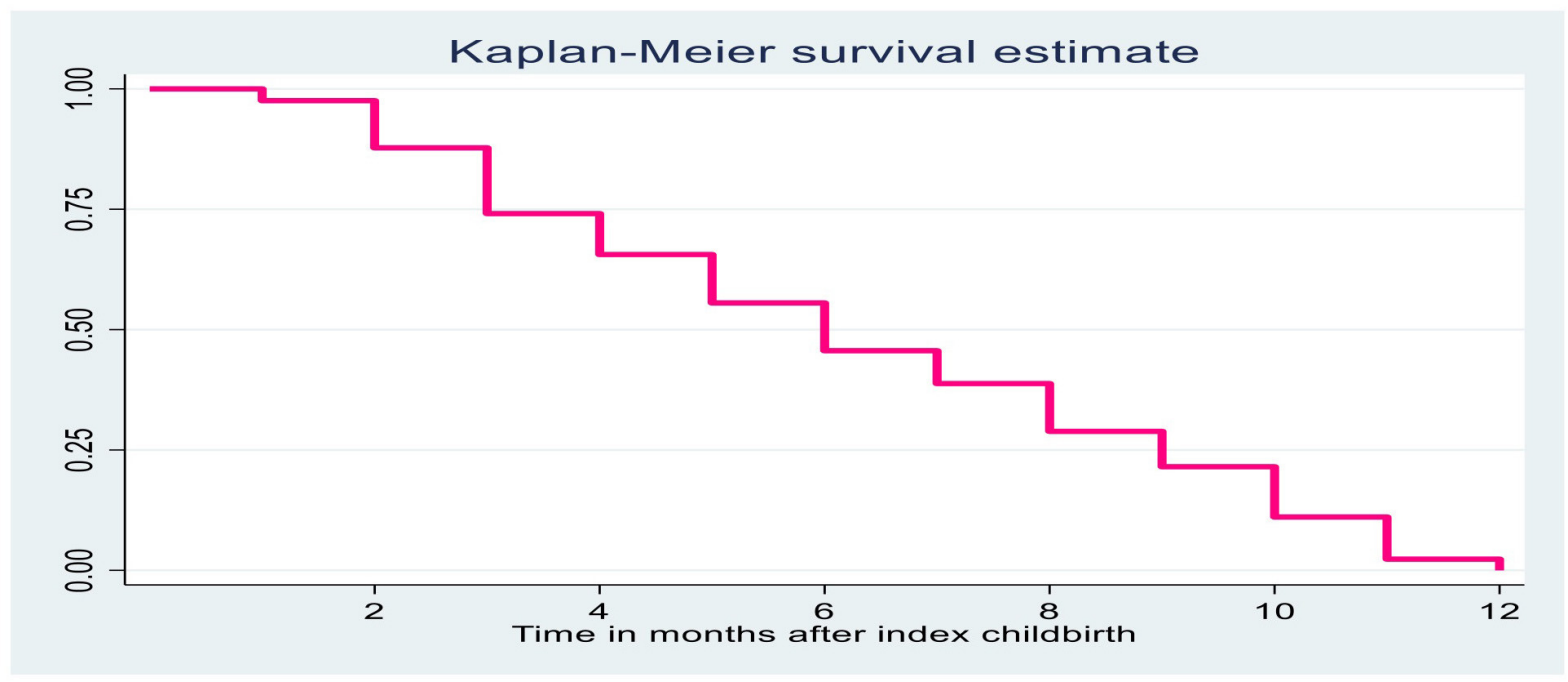
Kaplan Meier survival function curve for postpartum contraception timing after index childbirth among married women.

A total of 4,879 woman-months (407 women-years) were at risk of initiating modern contraception after index childbirth (Figure 1). The restricted mean survival time-to-first postpartum contraception was 6.28 months (95% CI: 6.07–6.51). At the 3 and 6 months postpartum, about 12.23 and 44.5% of the study participants had started their first modern methods, respectively. The Kaplan-Meier survival curves with large steps for time-to-postpartum contraceptive adoption start at 2 months postpartum. This indicates that many of postpartum women had started their first methods after 2 months.

**Figure 2:**
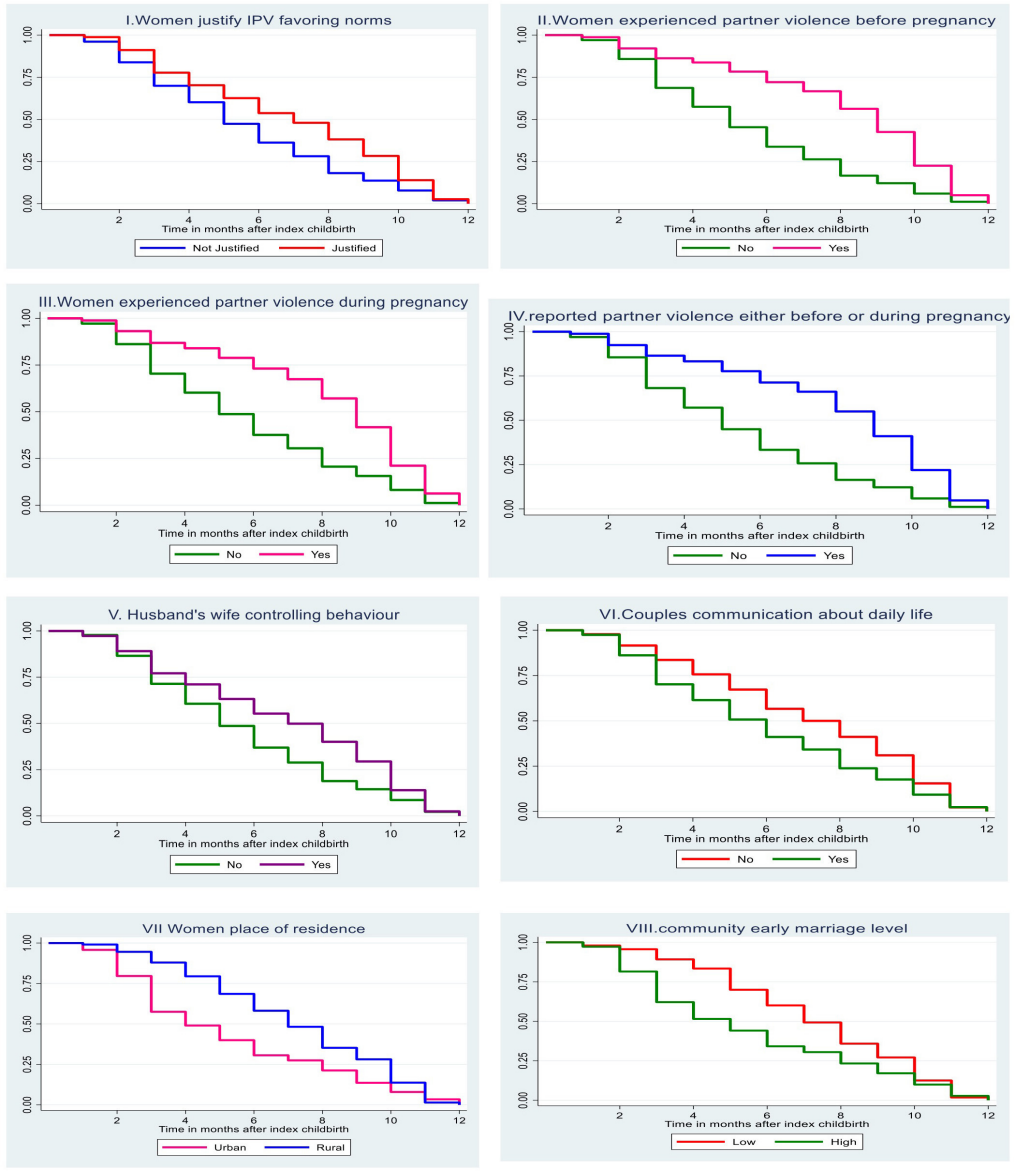
Kaplan-Meier estimate curves for postpartum contraception timing between index childbirth and 12 months by selected characteristics among married women.

We examined postpartum modern contraception timing for selected characteristics using the Kaplan-Meier survival estimate (Figure 2). Kaplan-Meir survival curve indicates a substantial difference in postpartum contraceptive method adoption between women who experienced PIPV and women who did not experience PIPV. The Kaplan-Meier survival function for women who experienced perinatal violence is consistently higher than their counterparts revealing that violence exposure before or/and during pregnancy lengthens time duration to modern methods adoption. In addition, Wilcoxon log-rank test has shown a significant difference in the length of survival time-to-postpartum contraceptive use at individual and cluster-level characteristics (Table 3).

**Table 3:** Wilcoxon log-rank test for the length of time-to-postpartum contraception use among married women.

**Variables**	**χ2**	***P*-value**	**Variables**	**χ2**	***P*-value**
Women's age in years	12.37	<0.0021	Exposure to PIPV in either periods	103.79	<0.00001
Women's age at marriage	14.37	<0.0001	Husband wife controlling behavior	25.13	<0.00001
Women's education Status	37.55	<0.0001	Husband's substance abuse	5.09	<0.0240
Women's attitude toward IPV norms	28.54	<0.00001	Husband's alcohol misuse	8.83	<0.0030
Women's decision-making autonomy	4.76	<0.03	Couple communication about daily life	17.65	<0.0001
Women's household wealth index	21.39	<0.00001	Respondent place of residence	42.73	<0.00001
Exposure to PIPV before pregnancy	104.41	<0.00001	Community's early marriage level	27.89	<0.00001
Exposure to PIPV during pregnancy	77.72	<0.00001	Community's IPV accepting status	15.79	<0.0001

### Model comparison for different parametric regression models based on the Akaike information criterion

We fitted different parametric survival models with different survival distribution for model selection: Exponential, Weibull, Gamma, Log-logistic, and lognormal. Weibull regression model was found to be the best-fitted model (Table 4). The ICC for the null model was computed using the variance of level-1 residual and variance of level-2(Keble) to identify the need of multilevel analysis (Table 5). The variance of the level-1(women) residuals is assumed to be independent and identically distributed, and their distributions depend on the model we are fitting. In the case of the Weibull distribution, the error term (residual) follows Gumbell distribution. We calculated residual variance using equation π^2^/(6 × ρ^2^), where ρ is the ancillary parameter of the Weibull distribution (52). ICC was found to be 0.805 indicates that 80.5% of the time length-to-postpartum contraceptive use can be explained by at cluster-level variance. In addition, the LR test was significant, which favored the multilevel Weibull regression model than standard Weibull model. Based on Akaike's Information Criterion (AIC), the full model was the most appropriate model that yielded the lowest deviance and AIC value, and selected to describe time-to- first postpartum contraceptive adoption.

**Table 4:** Model comparison parameters.

**Parameter**	**Deviance**	**AIC**	**BIC**
Exponential regression	4,355.93	4,395.93	4,489.02
Weibull regression	3,711.14	3,755.14	3,857.54
Gamma regression	3,723.96	3,767.965	3,870.35
Lognormal regression	3,767.85	3,811.853	3,914.24
Log-logistic regression	3,754.88	3,798.88	3,901.27

**Table 5:** Multivariable multilevel survival analysis of the postpartum contraceptive use timing among married women (*n* = 776).

**Characteristics**	**Categories**	**Model I**	**Model II**	**Model III**	**Model IV**
		**aTR [95% CI]**	**aTR [95% CI]**	**aTR [95% CI]**	**aTR [95% CI]**
**Community-level factors**					
Early marriage status	Low	na	na	Ref.	Ref.
	High	na	na	1.09 (0.98–1.21)	1.14 (1.01–1.28)^*^
Norm that favors IPV	Low	na	na	0.96 (0.87–1.06)	0.98 (0.88–1.10)
	High	na	na	Ref.	Ref.
Women education Level	Low	na	na	0.96 (0.84–1.09)	1.05 (0.91–1.21)
	High	na	na	Ref.	Ref.
Place of residence	Urban	na	na	Ref.	Ref.
	Rural	na	na	1.20 (1.03–1.38)^*^	1.44 (1.06–1.99)^*^
**Woman-level factors**					
Age at marriage	<18 Years	na	1.05 (0.97–1.12)	na	1.04 (0.96–1.12)
	≥18 Years	na	Ref.	na	Ref.
Maternal education	No formal	na	1.07 (0.97–1.18)	na	1.06 (0.96–1.17)
	Primary	na	0.98 (0.90–1.08)	na	0.98 (0.89–1.07)
	Secondary +	na	Ref.	na	Ref
Employment status	Not employed	na	Ref	na	Ref.
	Employed	na	0.95 (0.86–1.05)	na	0.97 (0.88–1.08)
Justify wife beating	No	na	Ref.	na	Ref
	Yes	na	1.02 (0.95–1.09)	na	1.01 (0.94–1.08)
Violence in either periods	No	na	0.72 (0.66–0.78)^***^	na	0.71 (0.66–0.78)^***^
	Yes	na	Ref.	na	Ref.
Wealth Status	Poor	na	0.97 (0.88–1.07)	na	0.98 (0.89–1.08)
	Middle	na	1.10 (1.02–1.20)^**^	na	1.10 (1.02–1.19)^*^
	Rich	na	Ref.	na	Ref.
DM autonomy	No	na	Ref.	na	1.01 (0.92–1.11)
	Yes	na	0.93 (0.86–0.99)^*^	na	Ref.
**Partner-level factors**					
Husband education	No education	na	0.99 (0.91–1.09)	na	0.96 (0.87–1.06)
	Primary	na	1.03 (0.94–1.12)	na	1.02 (0.94–1.11)
	Secondary +	na	Ref.	na	Ref.
Husband alcoholism	No	na	0.98 (0.92–1.06)	na	0.99 (0.92–1.06)
	Yes	na	Ref.	na	Ref.
Husband substance abuse	No	na	0.98 (0.88–1.06)	na	0.97 (0.88–1.07)
	Yes	na	Ref.	na	Ref.
Controlling behavior	No	na	Ref.	na	1.06 (0.93–1.1.08)
	Yes	na	0.99 (0.93–1.07)	na	Ref.
**Relationship-level factors**					
Couple communicate DLI	No	na	1.07 (0.99–1.15)	na	1.07 (0.99–1.15)
	Yes	na	Ref.	na	Ref.
**Random effects**					
lnp [Ancillary parameter]	–	2.274	0.894	0.803	0.892
Variance	–	0.072	0.126	0.038	0.083
LR-test(chi-square test)	LR test vs. Weibull model	16.03^*^	41.33^*^	9.83^*^	25.62^*^
Deviance (df.)	– 2LL	–	– 1860.696 (18)	– 1920.184 (7)	– 1855.573 (22)
Model statistics	AIC	–	3757.393	3854.368	3755.146
	BIC	–	3841.168	3886.947	3857.537
Heterogeneity level	ICC	0.81	0.31	0.10	0.23

* *P*-value < 0.05,

** *p*-vale ≤ 0.01,

*** *p*-value < 0.001, aTR, adjusted time ratios; ref, reference group; na, not applicable; DM, decision-making; DL, daily life issues; LR, likelihood Ratio.

### Multivariate multilevel survival analysis for time duration-to-the first modern contraceptive adoption among postpartum women

After controlling for other covariates, place of residence, community early marriage status, household economic status, and history of perinatal abuse were found to be predictors for the length of time-to-first modern contraceptive adoption (Table 5). Women from the rural community took 44% expected longer time to adopt the first postpartum modern contraception compared to women from the urban community (aTR: 1.44; 95% CI: 1.06–1.99). Similarly, women from the community with high early marriage had a 14% lag time to first postpartum modern contraceptive use compared to women from the community with low early marriage (aTR:1.14; 95% CI: 1.01–1.28). Besides, we have also examined the impact of perinatal partner abuse exposure on the postpartum contraceptive adoption. Women who reported no partner abuse before or/and during pregnancy had 29% expected lesser time to start the first postpartum modern contraceptive method than women who experienced PIPV (aTR: 0.71; 95% CI: 0.66–0.78). Women from the middle wealth quintiles were taken 1.10 times longer time to initiate postpartum contraception compared to women from the richest wealth quintiles (aTR: 1.10; 95% CI: 1.02–1.19).

## Discussion

This study has examined the individual- and community-level factors that predict the time duration of the first modern contraceptive adoption after childbirth, considering Keble as a cluster-level effect. The study found statistically significant heterogeneity in time interval-to-start modern methods across clusters. This finding indicates the influence of unobserved community-level characteristics, and is consistent with other studies that identified a woman's environment affects the timing of method use after childbirth (35). This implies the importance of leveraging community-level differences in planning intervention for timely modern contraceptive initiation. In the current study, 62.0% of women started using the first modern contraception in the year postpartum [95% CI: 59.1–64.5%]. This finding is consistent with studies conducted in Ethiopia (59.1%) and Kenya (60.0%) (35, 38). However, this estimate is considerably lower than other study done in Northwest Ethiopia (66.7%) (36) and higher than the nation-wide study done in Tanzania (37) and Ethiopia (56). We speculate that there may be differences in sample population characteristics, study design, and outcome variable measurement. For example, these nation-wide studies measured contraceptive adoption timing from the resumption of sexual intercourse, whereas our study examines the time length from delivery to the uptake of modern contraception. In this study, women's median survival time to start first postpartum modern contraception was found to be 6.3 months. This finding concurs with a study done in Northwest Ethiopia (36), but with at least 5 months' time lag than the WHO recommended time. This imply a sizeable proportion of postpartum women would be at risk of unintended pregnancy as many marks menses return to start contraception and requires community-based intervention during the perinatal and postpartum period.

Women's place of residence and their community early marriage status were predicted time interval-to-contraceptive use after childbirth when other variables were controlled for. As in other studies (56, 57), rural women took a long time to adopt modern methods postpartum than their counterparts. Our findings contrast somewhat with several prior studies (35, 37) that have identified no difference in time to modern contraceptive use among rural and urban residents. This might be correlated with rural women's limited access to media outlets, education, and health facilities infrastructure compared to urban women, which may also be associated with delayed adoption of modern contraception. This study was observed a long lag time to start contraception after childbirth among women from the community with high early marriage. This finding corroborates previous studies (58–60) that have identified early marriage is associated with a lower intention for postpartum contraceptive use. According to this study, women from middle wealth quantiles took longer time to start methods postpartum than women from the wealthiest households. This finding aligns with studies conducted elsewhere (37, 43) that show a shorter time to methods adoption among women in richest quintiles. The fact that low socioeconomic status is a deterrent to postpartum contraception adoption indicates a strengthening of social and community-based health insurance schemes launched by the government of Ethiopia, which increase health-care utilization among the poor (61). Besides, women empowerment could alleviate indirect costs like transportation for contraceptive use among low-in-come mothers even if contraceptive services are provided free of charge. Moreover, a quasi-experimental study by Deborah Sitrin et al. reported that integrating postpartum family planning into a health extension program could increase postpartum adoption of modern contraception (62).

There is inconsistency in the evidence regarding the association between intimate partner violence and the time interval-to-postpartum contraceptive initiations. While our finding confirms that women who had no history of perinatal abuse took less time to adopt modern contraception than those who had a history of abuse. For instance, Marina Plesons' prospective cohort study in Kenya shows a positive correlation between recent partner abuse and time to postpartum contraceptive adoption (63). As such, women's exposure to perinatal abuse may influence postpartum contraception timing in different ways: an abusive partner may restrict access to any form of contraception or prevent from using the most effective methods in an attempt to get the woman pregnant again (64, 65). Moreover, woman's less decisive power and fear of future violence linked with contraceptive initiation could deter timely adoption of the method after childbirth (66). This would imply intimate partner violence should be part of family planning counseling to identify a woman in a violent relationship which could significantly reduce the likelihood of future reproductive coercion.

In this community-based prospective study, applying the Weibull AFT model rather than the PH model to estimate expected survival times between group characteristics in time ratios may be the study's strength because estimated regression parameters in AFT models are robust and easy for interpretation of results. In addition to this significant strength, the finding should be interpreted with caution. The study had a limited follow-up period and frequency of interviews, which could be problematic given the persistence of protective factors like postpartum amenorrhea and abstinence. As this study is based on women's self-reported data collection methods, partner-characteristics and -controlled contraception may be underrepresented. A recent study did not address the timing of the contraceptive method mix. Although traditional methods are an important part of the pathways to avoid unwanted pregnancy, the scope of this study is very limited to identify traditional method users.

## Conclusion

In conclusion, rural residence, low household wealth status, and a high rate of early marriage in the community are predicted to lengthen the time to start modern contraception methods. In addition, a woman who had a history of violence either a year before or during pregnancy took a longer time than their counterparts to adopt modern contraception after childbirth. Thus, community-level women's empowerment, particularly among rural women and integration of intimate partner violence screening program into family planning counseling throughout the continuum of care will likely to improve postpartum contraception timing.

## Data availability statement

The raw data supporting the conclusions of this article will be made available by the authors, without undue reservation.

## Ethics statement

The studies involving human participants were reviewed and approved by Institutional Review Board of College of health sciences, Addis Ababa University. Written informed consent to participate in this study was provided by the participants' legal guardian/next of kin.

## Author contributions

TA has conceived the study, developed the proposal, conducted data collection and analysis, and drafted the manuscript. FG was involved in proposal development, fieldwork planning, and the result section. ND was involved in the proposal, data analysis and writing up and critical reviewing of the manuscript. All authors contributed to the article and approved the submitted version.

## Funding

Addis Ababa University, College of Health Sciences provided financial support for the data collection. It is a public university in Ethiopia and grants small amounts of money to Ph.D. candidates for data collection. Therefore, the university has no conflicts of interest in this study. No other funding was obtained for the current study.

## Conflict of interest

The authors declare that the research was conducted in the absence of any commercial or financial relationships that could be construed as a potential conflict of interest.

## Publisher's note

All claims expressed in this article are solely those of the authors and do not necessarily represent those of their affiliated organizations, or those of the publisher, the editors and the reviewers. Any product that may be evaluated in this article, or claim that may be made by its manufacturer, is not guaranteed or endorsed by the publisher.
